# SKN-1 isoform-c is essential for *C. elegans* development

**DOI:** 10.17912/micropub.biology.001205

**Published:** 2024-07-03

**Authors:** Tripti Nair, Carmen M. Ramos, Chris D. Turner, Vandita Gorla, Marisa Gaglio, Sean P. Curran

**Affiliations:** 1 University of Southern California, Los Angeles, California, United States

## Abstract

The transcription factor SKN-1 in *Caenorhabditis elegans* is a critical regulator of various biological processes, impacting development, diet and immune responses, cellular detoxification, and lipid metabolism; thereby playing a pivotal role in regulating the health and lifespan of the organism. The primary isoforms of SKN-1 ( SKN-1 a, SKN-1 b, and SKN-1 c) exhibit distinct functions resembling mammalian Nrf transcription factors. This study investigates the specific role of the SKN-1 c isoform in development by generating mutants with targeted missense mutations in the *skn-1 c* and *skn-1 a* isoforms. The *skn-1 c Met1Ala* mutants, which replaces a start methionine with alanine, renders SKN-1 c non-functional while preserving other isoforms, produced inviable embryos, requiring a balancer chromosome for proper embryonic development. In contrast, *skn-1 a Met1Ala* mutants, which replaces the start methionine with alanine for this isoform, displayed normal embryonic development and hatching. Moreover, the data suggest that SKN-1 c plays a crucial role in embryonic development, as strains without maternally deposited SKN-1 c lead to embryos that are developmentally arrested. Together, these findings contribute to our understanding of SKN-1 c's specific role in influencing embryogenesis and development in *C. elegans.*

**Figure 1. SKN-1 isoform c is essential for embryonic development:**
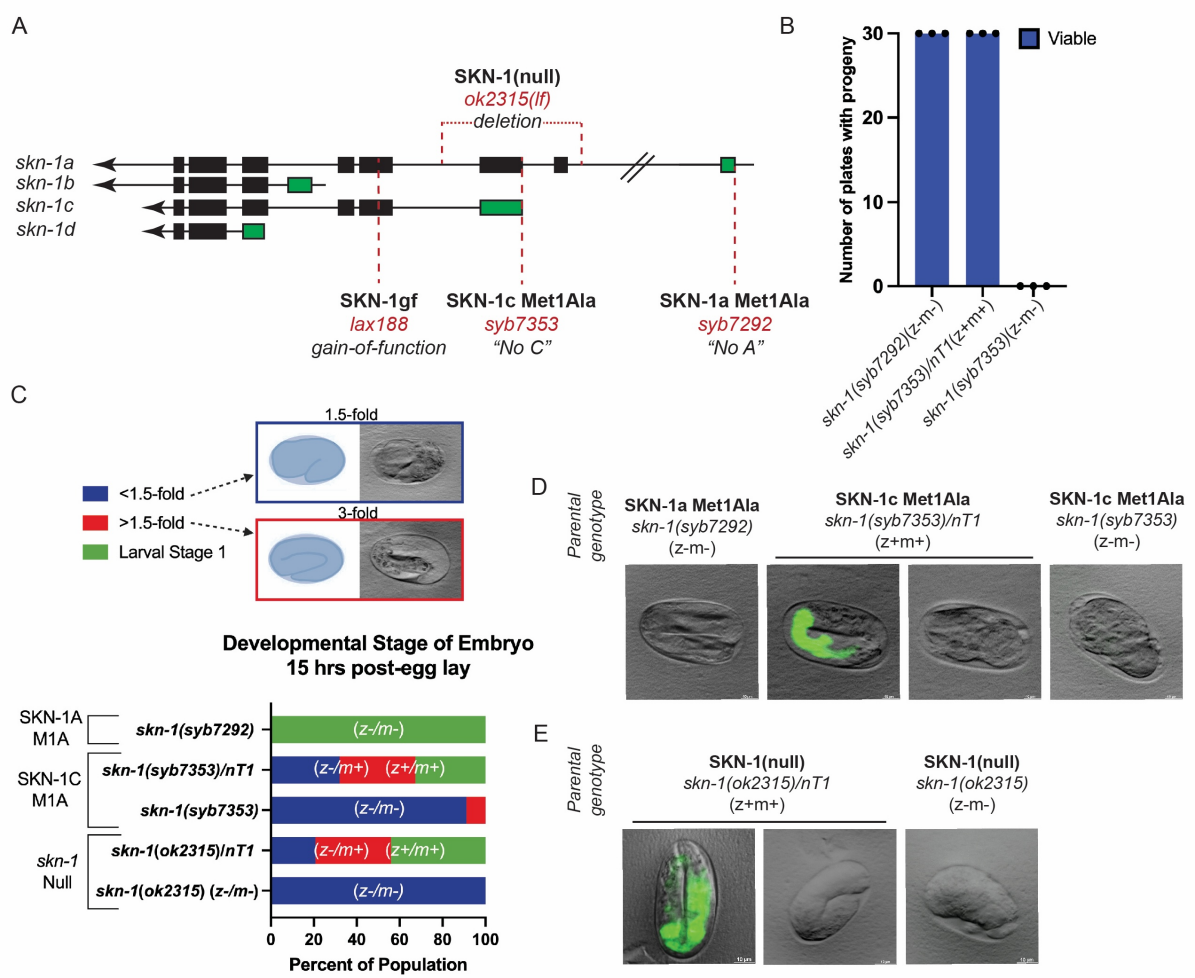
( **A** ) Schematic of *skn-1* isoforms marking the location of the *skn-1 a Met1Ala* (“no A”) and *skn-1 c Met1Ala* (“no C”) mutations. ( **B** ) Quantification of percentage of plates that have viable progeny on them after 72 hours. ( **C** ) Quantification of embryonic development of embryos 15 hours post-egg lay. Percent of population categorized into 3 groups: less than 1.5-fold (blue), greater than 1.5-fold (red), and larval stage 1 (green). ( **D-E** ) Imaging of embryos derived from parents of the indicated genotypes ( *skn-1 a Met1Ala, skn-1 c Met1Ala, or skn-1 ( ok2315 )* ); *nT1* balancer harbors an integrated GFP marker to follow presence of the element.

## Description

In *Caenorhabditis elegans* , the transcription factor SKN-1 plays a pivotal role in regulating various biological processes, including development, oxidative stress responses, detoxification, and lipid metabolism, thereby modulating health and lifespan (Blackwell, Steinbaugh et al. 2015) . SKN-1 has three primary functional isoforms in *C. elegans* generated through alternative splicing of the *skn-1* gene: SKN-1 a, SKN-1 b, and SKN-1 c. Each isoform has distinct functions and regulatory mechanisms that overlap with members of the mammalian Nrf transcription factor family, indicating the evolutionary conservation of “Cap'n'Collar” transcription factors. Consequently, *C. elegans* serves as a valuable model organism for understanding Nrf biology (Blackwell, Steinbaugh et al. 2015) .

During embryogenesis, SKN-1 is localized within the nuclei of intestinal precursor cells, while in postembryonic developmental stages, intestinal SKN-1 is predominantly cytoplasmic and accumulates in nuclei in response to both endogenous and exogenous stressors. Notably, the expression of intestinal SKN-1 predominantly consists of the SKN-1 a and SKN-1 c isoforms (Blackwell, Steinbaugh et al. 2015) . Among these isoforms, SKN-1 a is the longest and most extensively studied, playing a crucial role in regulating the expression of genes involved in oxidative stress response, proteasomal stress, detoxification, and lifespan regulation (Glover-Cutter, Lin and Blackwell 2013) . SKN-1 a isoform contains a transmembrane domain that associates with the endoplasmic reticulum (ER) membrane. Upon proteasome stress, SKN-1 a is released from the ER and upregulates the expression of proteasome machinery genes (Ruvkun and Lehrbach 2023) . On the other hand, SKN-1 c is primarily involved in cytoprotective responses (Blackwell, Steinbaugh et al. 2015) . While the specific functions of SKN-1 b remain less characterized compared to SKN-1 a and SKN-1 c, studies suggest that SKN-1 b is localized to ASI sensory neuronal pairs (ASIL and ASIR), and roles in caloric restriction-mediated longevity and oxidative stress resistance have been discussed (Bishop and Guarente 2007, Tullet, Hertweck et al. 2008, Tataridas-Pallas, Thompson et al. 2021) . More recently, studies on a constitutively active SKN-1 mutant ( *lax188* gain-of-function) that only alters the SKN-1 a and SKN-1 c polypeptide, but not SKN-1 b, was found to have a restricted expression in ASI neurons and this gain-of-function activity was sufficient to drive oxidative stress resistance (Turner, Stuhr et al. 2023) . The diverse isoforms of SKN-1 likely contribute to the versatility and specificity of its regulatory functions, potentially interacting with different sets of target genes or responding differently to various environmental stimuli. Understanding the functions and regulatory mechanisms of each isoform is crucial for unraveling the complexities of SKN-1-mediated processes in *C. elegans* .

The loss-of-function (lf) alleles of *skn-1* (e.g., *zu67 , zu129 ,* and *zu135 ,* and *ok2315* ) each result in a truncated protein and display a failure in the differentiation of the EMS blastomere into the appropriate tissues, resulting instead in differentiation into additional body wall muscle and hypodermis (Bowerman, Eaton and Priess 1992, Bowerman, Draper et al. 1993) . SKN-1 binds downstream partners *med-1* and *med-2* , which in turn bind to other associated differentiation factors, thereby determining the fate of the daughter cell in becoming the MS or E blastomere (Maduro, Meneghini et al. 2001) . SKN-1 is a maternally deposited mRNA for a tissue specification factor, playing a crucial role in the differentiation of the EMS blastomere into the MS and E cells during *C. elegans* embryogenesis; this differentiation is essential for the subsequent organogenesis of the pharynx and intestine, respectively (Bowerman, Eaton and Priess 1992, Bowerman, Draper et al. 1993) . More specifically, zygotic null mutants survive because of the maternally deposited *skn-1 (lf) (z-/m+* ) *,* whereas *skn-1 (lf) z-/m-* result in embryonic defects where the embryo fails to develop. In parallel, mammalian Nrf1 has been observed to be essential for development in mice. Nrf1 plays a role in the development of the hepatocyte lineage, as evidenced by abnormalities in liver development in *Nrf1−/−* mice (Chen, Kwong et al. 2003) . While the essential role of SKN-1 in intestinal development is evident, what remains unknown is the specific isoforms involved and their contributions to gut organogenesis and overall embryonic development. The functions of all SKN-1 isoforms are ablated in *skn-1* loss-of-function alleles *zu67 , zu129 ,* and *zu135* , complicating our understanding of which isoform(s) contribute to the development process. Based on the pivotal role played by SKN-1 in embryonic and postembryonic development, using both balanced and non-balanced genetic null mutants for each isoform will be informative in understanding which isoform(s) are responsible for intestinal specification and development.

Previous studies have observed vulval degeneration and a reduction in lifespan of balanced animals containing the *zu67* allele, which harbors mutations affecting both SKN-1 a and SKN-1 c; a phenotype attributed to the loss of SKN-1 a (Lehrbach and Ruvkun 2019) . However, a specific role for SKN-1 c in the context of development remains unknown. Here, to delineate the individual contributions of SKN-1 c, we generated mutants that contain a methionine to alanine missense mutation at the first methionine in the *skn-1 c* isoform ( *skn-1 c Met1Ala ( syb7353 )* ) rendering SKN-1 c inert while preserving the function of the other isoforms, and a methionine to alanine missense mutation in the *skn-1 a* isoform ( *skn-1 a Met1Ala ( syb7292 )* ) that maintains SKN-1 c structure ( **Figure 1A** ). The essential nature of the SKN-1 c isoform was observed as the *skn-1 c Met1Ala* mutants when propagated as homozygotes generate inviable embryos, phenocopying *skn-1 (lf)* alleles (Bowerman, Eaton and Priess 1992) and required a balancer chromosome ( *nT1* ) to maintain appropriate embryonic development. To confirm this observation, we singled 30 homozygous *skn-1 c Met1Ala ( syb7353 )* animals and 30 *skn-1 c Met1Ala/ nT1* animals and scored plates as either having viable larvae (post-embryonic development) or having no larvae on the plate. We scored plates three days after singling adults and confirmed that no progeny arose from any *skn-1 c Met1Ala* homozygous hermaphrodites, while all *skn-1 c Met1Ala/ nT1* animals laid viable embryos that developed normally and proceeded to post-embryonic development ( **Figure 1B** ). Conversely, the *skn-1 a Met1Ala* mutants displayed no evidence of embryonic lethality; generating viable embryos that developed and hatched ( **Figure 1B** ).

Although *skn-1* is essential for embryonic development, *skn-1 (lf)* mutants can be maintained by maintaining animals harboring the *nT1* balancer chromosome, and animals lacking zygotic (z) but receiving maternally (m) deposited *skn-1* will develop into fertile adults, but the ( *z-m-* ) embryos fail to develop. Previous examinations of *skn-1* null mutants by Bowerman et al., in 1992 demonstrated morphological defects in *skn-1* ( *z-m-* ) embryos, noting specifically the inability to progress to more advanced embryonic stages, where elongation occurs as the embryos undergo the morphogenesis of larval structures, such as the pharynx and intestine (Bowerman, Eaton and Priess 1992) . To characterize embryonic morphology in animals lacking either SKN-1 a or SKN-1 c, we assessed embryonic development in progeny from adults of the following genotypes: *skn-1 a Met1Ala* *(z-m-), skn-1 c Met1Ala/ nT1 (z+m+), skn-1 c Met1Ala (z-m-)* and compared these findings to *skn-1 ( ok2315 )/ nT1 (z+m+)* , and *skn-1 ( ok2315 ) (z-m-).* Embryos of each genotype were assessed and imaged by DIC microscopy 15 hours post-egg lay ( **Figure 1C -E)** . As expected, all embryos laid by *skn-1 a Met1Ala* animals hatched after 15 hours ( **Figure 1C,D** ). In contrast, ~70% of the embryos carrying the *nT1* balancer from *skn-1 c Met1Ala/ nT1* parents advanced beyond the 1.5-fold stage or hatched, and the remaining did not advance beyond the 1.5-fold stage; likely inviable *nT1 / nT1* homozygotes ( **Figure 1C,D** ). ~90 percent of the embryos from homozygous *skn-1 c Met1Ala* parents lacking maternal deposition of *skn-1* ( *z-m-* ) failed to progress past the 1.5-fold stage; with the remaining 10 percent progressing past the 1.5-fold stage but failing to hatch ( **Figure 1C,D** ). These findings are similar to embryos derived from *skn-1 ( ok2315 )/ nT1* animals where after 15 hours, 75 percent of the embryos were past the 1.5-fold stage or hatched with the remaining 25 percent arrested at the 1.5-fold stage (likely inviable due to *nT1* homozygosity), and 100 percent of *skn-1 ( ok2315 )* that did not receive maternal *skn-1 (z-m-)* arrested, similar to the *skn-1 c Met1Ala* *(z-m-)* embryos ( **Figure 1C -E** ).

Collectively, our data are consistent with a model in which SKN-1 c, but not SKN-1 a, is required for embryonic development, whereby embryos lacking maternally deposited SKN-1 c undergo developmental arrest. Taken together, these findings fill a gap in our understanding of the specific role for the SKN-1 c isoform in influencing embryogenesis. Further research to delineate the roles that each SKN-1 isoform plays in the regulatory networks that governing cellular functions with age will be critical for our complete understanding of the SKN-1 homeostat.

## Methods

***C. elegans* Strains and Maintenance**

*C. elegans* were raised on 6 cm nematode growth media (NGM) agar plates supplemented with streptomycin and seeded with *E.coli* strain OP50 . All strains were grown at 20°C.

We commissioned SunyBiotech to generate missense mutations in the initiator methionine, converting it to alanine, for the *skn-1 a* and *skn-1 c* isoforms in N2 Bristol, by CRISPR/Cas9 genome editing. The following strains were used:

SPC614 [ *skn-1* ( *syb7292* )] “ *skn-1 a Met1Ala”* . Superficially wildtype strain that contains a methionine to alanine missense mutation in the *skn-1 a* isoform.

SPC615 [ *skn-1* ( *syb7353* / *nT1* [ *qls51* ])] “balanced *skn-1 c Met1Ala* ”. Strain contains a methionine to alanine missense mutation in the *skn-1 c* isoform harboring the *nT1* balancer chromosome.

VC1772 *skn-1* ( *ok2315* ) IV/ *nT1* [ *qIs51* ] (IV;V) was received from the CGC.

**Fertility**

Thirty L4 stage animals of each genotype were singled onto individual NGM plates seeded with OP50 bacteria and allowed to propagate at 20°C. Plates were scored for the presence of progeny after 72 hours.

**Embryonic Development**

Three gravid adults were transferred to a fresh NGM plate with a small amount of OP50 bacteria to allow for better embryo visualization. Animals were allowed to lay eggs for six hours and the number of embryos were then recorded. After 24 hours, the embryos were examined to determine the number that had successfully completed development by hatching.

For embryonic and post-embryonic development assays, gravid adults (N=5, n=30) of each strain were transferred to a fresh NGM plate with a small amount of OP50 bacteria for 3-hours and then removed from the plates. The number of eggs laid were counted on each plate and evaluated again after 15 hours to assess development at the following embryonic stages (gastrulation, <1.5-fold, >1.5-fold, or hatched larva).

**Imaging**

The total number of eggs was counted using a Nikon SMZ 800. The population studies for the ex-utero development were performed using the Leica M205 microscope. Live eggs were observed and imaged at a total magnification of 62.8x ≈ 63x with LAS X software via the Leica M205 microscope and Leica KI5 camera.
